# Lipoprotein-Specific Fatty Acid Profiles in Familial Hypercholesterolemia: Associations with Cardiovascular History and Dietary Patterns

**DOI:** 10.3390/nu18010092

**Published:** 2025-12-27

**Authors:** Anallely López-Yerena, Raquel Arroyo-Olivares, Victoria Santisteban, Natalia Muñoz-Garcia, Ramón Estruch, Pedro Mata, Lina Badimon, Teresa Padro

**Affiliations:** 1 Institut Recerca Sant Pau (IR Sant Pau), Sant Quintí, 77-79, 08041 Barcelona, Spain; nayelopezye@ub.edu (A.L.-Y.); vsantisteban@santpau.cat (V.S.); nmunoz@santpau.cat (N.M.-G.); lbadimon@ficsi.org (L.B.); 2 Department of Internal Medicine, Hospital Clínic, Institut d’Investigació Biomèdica August Pi i Sunyer, Universitat de Barcelona, 08036 Barcelona, Spain; restruch@ub.edu; 3 Centro de Investigación Biomédica en Red de Fisiopatologia de la Obesidad y la Nutrición (CIBEROBN 0603), Instituto de Salud Carlos III, 28029 Madrid, Spain; 4 Institut de Recerca en Nutrició i Seguretat Alimentaria (INSA-UB), University of Barcelona, 08921 Barcelona, Spain; 5 Fundación Hipercolesterolemia Familiar, 28010 Madrid, Spain; fhfdieta@colesterolfamiliar.org (R.A.-O.); pmata@colesterolfamiliar.org (P.M.); 6 Centro de Investigación Biomédica en Red Cardiovascular (CIBERCV), Instituto de Salud Carlos III, 28029 Madrid, Spain; 7 Medical School, Universitat de VIC-UCC and IRSI-CC, Vic, 08500 Barcelona, Spain; 8 Cardiovascular Research Foundation for Health Prevention and Innovation (FICSI), 08017 Barcelona, Spain

**Keywords:** lipid metabolism, LC-MS/MS analysis, lipoproteins, DHA, EPA, arachidonic acid, Mediterranean diet

## Abstract

**Background/Objectives**: Familial hypercholesterolemia (FH) is an autosomal dominant inherited disease characterised by lifelong LDL cholesterol levels and premature presentation of cardiovascular disease if left untreated. Whether fatty acid (FA) composition in lipoproteins is modified in FH patients is not known. This study aimed to identify FA differences in low- and high-density lipoprotein (LDL and HDL, respectively) among young Spanish individuals with FH, treated as per guidelines recommendations, compared to their unaffected relatives with similar LDL concentrations in plasma. We also evaluated associations between the occurrence of cardiovascular event (CVE), dietary patterns, and the lipoprotein FA profile in FH. **Methods**: Lipoprotein FA profiles were determined by liquid chromatography/mass spectrometry/mass spectrometry (LC/MS/MS). **Results**: In comparison to their non-FH relatives, FH patients showed changes in the FA profile, predominantly in LDL particles while HDL particles were only modestly changed. FH individuals exhibited higher concentrations of poly- and monounsaturated FAs, oleic, γ-linoleic, α-linoleic, arachidonic, and eicosapentaenoic acids (*p* < 0.05). Interestingly, FH individuals showed greater adherence to the Mediterranean diet than their non-FH relatives, with no significant differences between those with and without previous CVE. The most pronounced changes in FA profile were observed in FH patients with a history of CVE, although the event itself did not significantly modify lipoprotein FA profiles. **Conclusions**: Well treated FH patients showed a FA profile that responded to a healthier diet than their relatives with similar plasma LDL levels. The strict lifestyle and pharmacological treatment affected positively the lipoproteins of FH patients and needs to be recommended.

## 1. Introduction

Familial hypercholesterolemia (FH) is an inherited condition associated with lifelong exposure to high circulating levels of low-density lipoprotein cholesterol (LDLc) [1,2], leading to an increased risk of premature atherosclerotic cardiovascular disease (ASCVD) [3,4,5]. In Spain, the number of people with FH is estimated to be 200,000 [6]. In most cases, FH is caused by mutations in the LDL receptor (LDLR) gene; however, FH patients with the same genetic background often exhibit significant phenotypic variability [7]. This heterogeneity is well documented in the national SAFEHEART (Spanish Familial Hypercholesterolemia Cohort Study) cohort, a large, multicenter, and prospective registry of FH patients in Spain [8], from which the present study population was drawn.

While the main feature in FH is the impact of high levels LDL cholesterol in atherosclerosis and ASCVD, metabolic phenotype and lipid type composition of both LDL and high-density lipoprotein (HDL) particles may also be altered in FH subjects, affecting their atherogenicity, through mechanisms beyond their cholesterol content [9,10,11].

Growing evidence in FH patients suggests that hypercholesterolemia may also perturb other metabolic processes, including those involving fatty acid (FA), amino acids, and glucose [12]. However, the underlying mechanisms remain poorly understood. It is known that the FA composition of lipoproteins is influenced by complex interactions involving FA and lipoprotein synthesis, conversion, and metabolism, as well as by dietary, pharmaceuticals, and pathological conditions [13].

Most studies have focused on the relationship between the fatty acid profile of total blood lipids and cardiovascular disease (CVD). However, the FA profiles of HDL and LDL are important determinants of their functional properties and CVD risk [14]. For LDL, higher polyunsaturated fatty acids (PUFAs)—particularly arachidonic acid—positively correlate with oxidation susceptibility and CAD, while monounsaturated (MUFA, oleic acid) and saturated fatty acids (SFA) confer resistance; small dense LDL enriched in SFA/MUFA exhibits greater atherogenicity due to arterial penetration and oxidation [15,16,17]. For HDL, phospholipid FA composition modulates cholesterol efflux and anti-inflammatory functions; unsaturated FA-enriched HDL (linoleic, omega-3) supports anti-atherogenic activity, while SFA/MUFA enrichment (palmitic, oleic, palmitoleic) with linoleic depletion characterizes dysfunctional HDL in metabolic syndrome and premature CAD; small dense HDL3 enriched in phosphatidylserine/phosphatidic acid enhances protective functions [14,18]. Regarding FA in FH, a cross-sectional study in children with FH reported higher plasma FA concentrations in those untreated with statins compared to controls; however, these observations were limited to plasma and did not include lipoprotein fractions [19]. More recently, lipidomic analyses in FH patients have revealed abnormal levels of triacylglycerols, diacylglycerols, and 7-ketocholesterol associated with heterozygous phenotype [20].

Statins are currently the primary treatment for lowering LDLc in FH patients, particularly those with heterozygous FH [21]. Preliminary evidence suggests that statins may also affect the synthesis and tissue composition of long-chain polyunsaturated fatty acid (PUFA). Thus, statins enhance the conversion of linoleic acid (LA) and eicosapentaenoic acid (EPA) into their long-chain derivatives [22,23]. However, despite the importance of FAs for health, little is currently known about the FA composition of HDL in FH patients; most studies have focused on assessing the composition of LDL.

Most studies have observed a similar lipid composition in LDL in FH patients and normolipidemic subjects [24,25,26]; however, these studies were conducted in countries with different lifestyles and dietary patterns to the Mediterranean diet (MedDiet), such as South Africa and Belgium, suggesting that the FA profile could differ in vulnerable populations such as the FH. In addition, the importance of the composition of FAs in lipoproteins is related to their role in maintaining normal lipoprotein functionality, including antioxidant properties [27,28].

In this study, we used a mass spectrometry-based lipidomics approach to profile the FA composition of LDL and HDL particles in individuals with FH and their unaffected relatives from the well-characterized SAFEHEART cohort. LDL and HDL were selected because they are the lipoproteins most strongly implicated in FH-related atherogenesis: LDL through impaired clearance and arterial lipid deposition, and HDL through its contribution to residual cardiovascular risk. Other lipoproteins, such as VLDL, although more lipid-rich, play a lesser role in FH due to generally normal tri-glyceride levels and their rapid metabolic conversion to LDL. Additionally, we investigated whether specific FA profiles were associated with the prevalence ASCVD in genetically confirmed FH cases, while accounting for adherence to the MedDiet.

## 2. Materials and Methods

### 2.1. Subjects

The study design and patient recruitment have been described previously [29]. The present study included 45 patients: 30 with clinical and genetic diagnosis of FH and 15 non-FH individuals, matched for demographics, all from the SAFEHEART cohort. Patients with FH were further classified based on their history of cardiovascular events (CVE), including angina pectoris, stroke, transient ischemic attack, acute myocardial infarction, and peripheral vascular disease, into two groups: those without CVE (FH/non-CVE, n = 15) and those with CVE (FH/CVE, n = 15). More detailed information regarding the definition of ASCVD in the SAFEHEART cohort has been previously described [30].

As shown in Table S1, FH patients in the study group (N = 30) were mostly treated with statins (>93%) for more than one year at the time of entering the study (atorvastatin: N = 22, simvastatin: N = 4, pravastatin: N = 2), in accordance current clinical practice guidelines. In addition, 76.7% FH patients received ezetimibe and 6.7% underwent LDL apheresis. Among non-FH participants (N = 15), 4 subjects were treated with statins (26% statins; simvastatin: N = 4, atorvastatin: N = 1, pravastatin: N = 1), 2 with fibrates (13.3%) and 2 with ezetimibe (13.3%). Regarding glucose-lowering medications, 3 FH participants were treated with antidiabetic drugs (sulfonylureas and/or metformin), and one non-FH was treated with insulin. Antihypertensive therapy was used in 6 FH and 2 non-FH and participants, including betablockers, calcium-channel blockers, ACE inhibitors, angiotensin II receptor blockers (ARBs), and diuretics.

Informed consent was obtained from all patients, and the local ethics committee of the Investigación Clínica Fundación Jimenez Diaz (protocol number: 01/09) approved the study, which was conducted according to the Declaration of Helsinki and an informed consent was obtained from all participants. 

### 2.2. Data Collection

Data collected on the demographic and clinical characteristics of the subjects included age, sex, CVD risk factors including other than hypercholesterolemia including hypertension, type 2 diabetes mellitus, smoking status, findings on physical examination, history of CVE, and current lipid-lowering treatment. Findings on physical examination included weight, height, body mass index (kg/m^2^), and waist circumference (WC, cm/cm). Blood pressure was measured twice with each subject in a supine position using an Omron MX3 sphygmomanometer (Mannheim, Germany).

On the day the samples were collected, participants were instructed to complete a food frequency questionnaire (FFQ) detailing their dietary habits. The FFQ used was validated in Spanish patients with FH [31]. Each participant received oral and written instructions to self-complete the FFQ. The dietary intake of saturated-, monounsaturated- and polyunsaturated FAs (SFA, MUFA, and PUFA, respectively) as well as the consumption of individual FAs was obtained.

The adherence to the MedDiet was calculated using the validated 14-item Mediterranean Diet Adherence Screener (MEDAS) used in the Prevencion con Dieta Mediterranea (PREDIMED) study [32].

### 2.3. Sample Collection and Biochemical Analysis

Venous blood was withdrawn after 10–14 h fasting from the cubital vein without tourniquet using a 20-gauge needle for all patients at entry in the SAFEHEART cohort. Blood samples were collected without anticoagulant for serum preparation or in ethylenediamine tetra-acetic acid (EDTA)-containing Vacutainer tubes for plasma preparation. Serum and EDTA plasma were collected for biochemical analysis under standardized conditions. All samples were processed identically, within the first two hours after extraction.

For LDL and HDL isolation, plasma was obtained at 1258× *g* for 20 min at room temperature [33]. Aliquots were generated from all plasma samples, snap-frozen in liquid nitrogen and stored at −80 °C until analysis.

Serum biochemical measurements were performed at a single laboratory [34]. For lipid profile, total cholesterol (TC), triglycerides (TAG), and HDLc were measured by standardized enzymatic methods and when TG levels were lower than 350 mg/dL, LDL cholesterol (LDLc) was calculated using Friedewäld formula [35].

The serum concentration of lipoprotein A [Lp(a)] was quantified using an isoform independent assay [Quantia Lp(a) 7K00-01; Tulip Diagnostics, Bambolim, India] and an Architect autoanalyzer C16000 (Abbott Diagnostics, Lake Forest, Illinois) that was calibrated using the International Federation of Clinical Chemistry (IFCC) reference apo(a) standard (IFCC/SRM 2B, https://pubmed.ncbi.nlm.nih.gov/11106328/ (accessed on 22 December 2025); Milan, Italy). Inter-assay variation was <7% [36].

### 2.4. Lipoprotein Sample Preparation

LDLs were obtained from 2 mL plasma-EDTA samples through successive ultracentrifugation steps within the density range of 1.019 to 1.063 g/mL, following the procedure initially outlined by Havel, Eder, and Bragdon [37]. Human HDL were obtained by ultracentrifugation in KBr gradient of EDTA plasma (density gradients 1.063–1.210 g/mL), and the protein fraction was obtained by precipitation with pure ice-cold acetone (protocol that enables the delipidation of HDL samples) and solubilized in a urea/thiourea buffer (7 M urea, 2 M thiourea, 2% CHAPS) [38].

### 2.5. LC/MS-MS

FA pattern was determined in LDL and HDL samples, as previously described by Padro et al. [39], and were analyzed by liquid chromatography/mass spectrometry/mass spectrometry (LC/MS/MS) using a HPLC system (Agilent 1200, Santa Clara, CA, USA) coupled to an AB Sciex 3200 Qtrap triple quadrupole MS (SCIEX, Framingham, MA, USA).

Analysis of the FA profile by LC/MS/MS was performed after derivatization of the samples to convert FAs in trimethylaminoethyl ester iodide derivatives. Briefly, lipoprotein samples (10 μL), internal standards (32 μL) and acetonitrile (360 μL) were hydrolizated in 40 μL of hydrochloric acid (5 M) at 100 °C for 1 h. Afterwards samples were extracted with hexane (1 mL) and derivatized with oxalyl chloride, dimethylaminoethanol and methyl iodide (100 μL). After 2 min at 25 °C, the mixture was evaporated, and the residue was dissolved in ethanol (500 μL).

FAs were separated using a LC system with a Zorbax Eclipse XDB-C18 column (4.6 × 50 mm, 1.8 μm particle size) and a binary gradient program consisting of 10 mM ammonium acetate in 40:60 acetonitrile/water *v*/*v* (mobile phase A), and 10 mM ammonium acetate in 10:90 acetonitrile/isopropanol (mobile phase B). The following gradient was used: 0 to 1 min, B = 40%, 1 to 11 min, B = 40 to 100%, 11 to 15 min, B = 100%, 15 to 18 min, B = 100 to 40%, and 18 to 20 min, B = 40%. The mobile phase was delivered at a flow rate of 0.4 mL/min and the analysis was performed at 55 °C using a column oven.

The mass spectrometer instrument was equipped with a turbo spray source operated in positive ionization mode. The source was operated at 400 °C with ion voltage of 5500 V, and nitrogen as curtain and nebulizer gas. For analysis of FA profiles, a neutral loss scan (NL) of 59.0 was used. For accurate quantification of specific targeted FAs, a multiple reaction monitoring (MRM) approach was performed, using the MS instrument with an ion spray and positive ion operating mode at 400 °C.

Triple quadruple mass spectrometry (QqQ-MS) data were analyzed with LipidView version 1.1 software (AB Sciex). Lipid identification was based on their characteristic head groups and corresponding FAs against the LipidView library, which contains over 50 lipid classes, 25,000 lipid species, and more than 600 characteristic lipid fragments. Quantification was conducted on the basis of an internal standard mixture added to the samples prior to the derivatization procedure: myristic-d27 acid (3.3 M), palmitic acid-1-13C (0.3 M), and stearic-2,2-d2 acid (1.3 M), from Sigma Aldrich (St. Louis, MO, USA), and 0.3 M arachidonic acid (AA) AA-d8 and 0.3 M LA-d4 from Cayman Chemical (Ann Arbor, MI, USA).

### 2.6. Statistical Methods

Statistical analyses were performed using STATA 15.1 (College Station, TX, USA) software. Firstly, the Shapiro–Wilk test was applied to verify the normal distribution of data. The non-parametric Mann–Whitney test or (χ^2^ test) was used to evaluate differences in anthropometrical measures and plasma lipids between non-FH subjects and total FH, FH-No CVE and FH-CVE: (i) differences in anthropometrical measures and plasma lipids between FH and non-FH subjects; (ii) differences in FA profile (SFAs, MUFAs, PUFAs and individual FAs) in LDL and HDL in FH and non-FH subjects; (iii) differences in FA profile in LDL and HDL between non-FH and FH/CVE, non-FH and FH/non-CVE, and FH/CVE versus FH/non-CVE. Data are expressed as median [IQR]. A two-sided *p* value < 0.05 was considered statistical significance.

## 3. Results

### 3.1. Participant Characteristics

Anthropometric, hemodynamic and biochemical variables are summarized in Table 1. Overall, 33% of the participants in the study were women, with an uneven distribution between groups (46.7% women in the non-FH and 26.7% women in the in the FH age group). Among women, 22% were postmenopausal (four women in the non-FH group and six women in the FH group; χ^2^ *p* = 0.390). Of the 45 subjects in the study, seven individuals (15.5%) were active smokers and 21 (24%) were ex-smokers at the time of entering the SAFEHEART cohort (χ^2^ *p* = 0.515).

The groups differed in age (non-FH: 44.5 [39.0, 58.0] years; FH: 59.0 [46.0, 64.0] years, *p* = 0.009). No significant differences were observed in weight, BMI, or WC (*p* > 0.05) between groups. Diastolic blood pressure (DBP) did not differ significantly, whereas systolic blood pressure (SBP) was higher in FH subjects (*p* = 0.018). Regarding the lipid profile or biochemical parameters, no statistically significant differences were found in levels of TAG, TC, HDLc, LDLc, ApoB, ApoA1, Lp(a), C-reactive protein (CRP) and glucose levels. Fasting glucose had median [IQR] levels in serum below 95 mg/dL in all groups. However, 4 of 45 subjects were diabetic, one subject in the non-FH group and three in the FH group (10%, 2 of them in the subgroup with clinical CVD).

Among the 30 individuals with FH, 15 had no history of CVE (FH/non-CVE), while the other 15 had a documented history of CVE (FH/CVE) at the time of entering the SAFEHEART cohort. When comparing the FH/non-CVE group with their unaffected relatives, no differences were observed in anthropometric, blood pressure, or lipid profile variables between the two groups. In contrast, subjects with FH and a history of CVE were older (FH/CVE: 61.0 [56.0, 64.0] years vs. non-FH: 44.0 [39.0–58.0] years; *p* = 0.001) and had higher levels of SBP (*p* = 0.004) than subjects without FH

Within the FH population, individuals with and without a history of CVE showed no statistically significant differences in anthropometric, blood pressure, or biochemical parameters.

### 3.2. Adherence to MedDiet and Dietary Fatty Acid Intake

**Figure 1:**
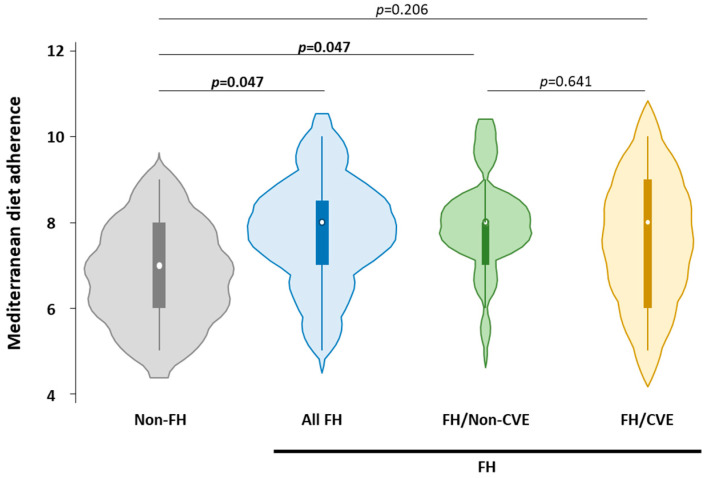
Mediterranean diet adherence among hypercholesterolemic and normocholesterolemic patients, including individuals with familial hypercholesterolemia, based on the presence or absence of cardiovascular events. FH: familial hypercholesterolemia; CVE: cardiovascular event. *p* < 0.05 was considered significant.

Figure 1 illustrates adherence to MedDiet calculated using the validated 14-item MEDAS score. Individuals with FH showed significantly higher adherence compared to non-FH subjects (all FH: 8.0 [7.0–8.5] vs. non-FH: 7.0 [6.0–8.0], *p* = 0.047).

When FH individuals were stratified by history of CVE, subgroups with and without CVE (FH/CVE and FH/non-CVE) exhibited a trend toward higher adherence than their non-FH relatives; however, only the FH/non-CVE group showed a statistically significant increase (FH/non-CVE: 8.0 [7.0–8.0] vs. non-FH: 7.0 [6.0–8.0], *p* = 0.047) in adherence. No significant difference in MedDiet adherence was observed between FH individuals with and without a history of CVE (*p* = 0.641), indicating a similar dietary pattern between these two groups.

Table 2 presents the intake of SFAs, MUFAs, and PUFAs, as well as individual FA consumption in subjects with FH and their unaffected relatives. Overall, FH and non-FH subjects showed similar intake of SFAs, MUFAs, and PUFAs, except for stearic acid intake, which was lower in subjects with FH (FH: 2.8 [2.2–4.5] g/day vs. non-FH: 3.7 [3.4–7.2] g/day, *p* = 0.027). Specifically, when individuals with FH who had not experienced a CVE were compared with their unaffected relatives, intake of stearic and arachidonic acids were significantly lower in the FH/non-CVE group. (FH/non-CVE vs. non-FH: *p* = 0.042 and *p* = 0.020, respectively). In contrast, FH individuals with history of CVE reported a higher intake of EPA and DHA compared to their healthy relatives (FH/CVE vs. non-FH: *p* = 0.007 and *p* = 0.012, respectively). Similarly, among individuals with FH, both EPA and DHA levels were significantly higher in the FH/CVE group compared to FH/non-CVE group (*p* = 0.034 and *p* = 0.040, respectively). It is worth noting that the diets of FH subjects showed a trend toward a lower content of trans fatty acids (*p* = 0.055), although this difference did not reach statistical significance.

### 3.3. Lipoprotein Fatty Acid Profile

Levels of SFAs, MUFAs, PUFAs, as well as individual FAs were assessed in both LDLs and HDLs fractions of FH individuals without and with history of CVE and in the non-FH relatives, and are given in Table 3 (LDL) and Table 4 (HDL). The relative abundance of these FAs in LDLs and HDLs is provided in Supplemental Tables S2 and S3, respectively.

#### 3.3.1. Differences Between FH Individuals and Non-FH Relatives

Individuals with FH exhibited LDL particles with significantly higher concentration of MUFAs (*p* = 0.049) and PUFAs (*p* = 0.032) than in non-FH subjects’ LDL, while SFA profile did not differ between FH and non-FH groups. Specifically, the LDL of FH patients contained higher levels of oleic acid (*p* = 0.049), γ-linolenic acid (*p* = 0.002), arachidonic acid (*p* = 0.003), α-linolenic acid (*p* = 0.040), and EPA (*p* = 0.008) (Table 3). Regarding the relative abundance of FAs in LDL (Table S2), the proportion of linoleic acid was 0.92-fold lower in FH subjects, whereas the relative abundance of γ-linolenic acid, arachidonic acid, and EPA were 1.2-fold, 1.3-fold, and 1.9-fold higher, respectively (all *p* < 0.05).

In HDL, subjects with FH displayed a distinct FA profile compared with non-FH individuals (Table 4). Subjects with FH had an HDL FA profile characterized by higher MUFA levels, particularly oleic acid (*p* = 0.003), and elevated PUFA species, including γ-linolenic acid (*p* = 0.013), arachidonic acid (*p* = 0.006), and EPA (*p* = 0.012), whereas SFAs remain comparable between groups. Regarding the relative abundance of FAs in HDL (Table S3), the proportion of linoleic acid was 0.76-fold lower in FH patients, whereas the relative abundance of γ-linolenic acid, arachidonic acid, and EPA were 1.2-fold, 1.3-fold, and 1.7-fold higher, respectively, in FH individuals (all *p* < 0.05).

#### 3.3.2. Differences Between FH Without CVE and Non-FH Relatives

Only a few differences in LDL-FA composition were revealed between FH without CVE and non-FH relatives (Table 3). Specifically, the LDLs of FH individuals without history of CVE were enriched in γ-linolenic (*p* = 0.019) and arachidonic acid (*p* = 0.041). However, when examining relative abundances, only the proportion of γ-linolenic acid was significantly higher in the FH/non-CVE group compared to its healthy relatives (FH/non-CVE: 7.7 [6.3–11.2] % vs. non-FH: 6.1 [4.4–7.0] %, *p* = 0.016, Table S2).

In HDL particles, FH/non-CVE individuals showed higher γ-linolenic acid levels, both in concentration (Table 4) and relative abundance (Table S3), and lower proportion of DHA compared to non-FH subjects (all *p* < 0.05).

#### 3.3.3. Differences Between FH with CVE and Non-FH Relatives

In LDL particles, FH patients with a history of CVE exhibited significantly higher concentration of oleic acid and PUFAs, including γ-linolenic acid, arachidonic acid, α-linolenic acid and EPA (all *p* < 0.05). No differences were observed in SFAs, linoleic acid and the *ω*6/*ω*3 ratio (Table 3). In contrast, analysis of relative FA abundances in LDL (Table S2) showed that FH/CVE individuals had lower proportions of linoleic acid and higher proportions of γ-linolenic acid, arachidonic acid, and EPA compared with non-FH subjects [fold changes FH/CVE vs. non-FH: 0.8, 1.2, 1.7, and 2.9, respectively, all *p* < 0.05].

In HDL particles, individuals with FH and CVE exhibited significantly higher concentrations of oleic acid and several PUFAs, including γ-linolenic acid, arachidonic acid, and EPA, compared to controls (all *p* < 0.05; Table 4). Consistently, in terms of relative abundance (Table S3), these PUFAs were also significantly elevated in the FH group. Conversely, the proportion of linoleic acid was significantly reduced (all *p* < 0.05).

#### 3.3.4. Comparison Within the FH Population Based on CVE History

The FA concentration of LDL particles in individuals with FH did not significantly differ based on the presence or absence of a CVE (Table 3). However, EPA that showed a trend toward higher levels (1.5-fold), while the *ω*6/*ω*3 ratio displayed a decreasing trend (0.78fold) in FH patients with history of CVE. When relative abundances were considered, the proportions of linoleic acid and EPA in LDL differed significantly between FH patients without and with history of CVE (fold changes FH/non-CVE vs. FH/CVE; linoleic acid: 1.25-fold, *p* = 0.005; EPA: 0.86-fold, *p* = 0.044; Table S2).

The FA concentration of HDL particles did not significantly differ between individuals with FH with or without a CVE (*p* > 0.05; Table 4). The only exception was the *ω*6/*ω*3 ratio, which was significantly higher in the FH without CVE group (*p* = 0.007). Regarding relative abundance (Table S3), DHA was significantly more abundant in individuals with FH and CVE compared to those without CVE (*p* = 0.013), while the relative levels of other FAs remained similar between groups.

## 4. Discussion

This study, conducted within the SAFEHEART cohort, presents the first comprehensive characterization of lipoprotein FA profiles in individuals with FH compared with their unaffected relatives, while also examining the influence of CVE history and adherence to the MedDiet. The study findings provide novel insights into alterations in lipoprotein FA composition in FH patients and suggest underlying disturbances in lipoprotein FA metabolism that may influence LDL and HDL functionality and contribute to the atherogenic processes associated with the presence of FH. Specifically, (i) FH is linked to distinct remodeling of lipoprotein FAs, predominantly in LDL particles, while HDL is only modestly affected; (ii) the most pronounced alterations compared to non-FH relatives were observed in FH individuals with a history of CVE; and (iii) within the FH population, a history of CVE does not appear to substantially modify these FA patterns.

LDL particles are more susceptible to changes in FA composition than HDL. Previous studies have shown that LDLs serve as sensitive biomarkers of alterations in FA metabolism due to their greater compositional plasticity and more direct regulation by dietary FAs at the gene expression level. In contrast, although HDL composition can also be influenced by FAs, its response tends to be less pronounced and more variable, depending on the type of FA involved [40,41,42].

In this study, LDL particles from individuals with FH were enriched in MUFAs and PUFAs. These variations are likely influenced by the higher adherence to the MedDiet observed in individuals with genetically confirmed FH. This observation is consistent with the descriptive analysis of the eating habits of the SAFEHEART cohort, in which the authors observed that patients with FH followed a healthier diet than their unaffected relatives. Specifically, individuals with HF and a history of CVD had the most favorable FA profile and greater adherence to the MedDiet [43].

Our results suggest that LDLs from FH individuals do not exhibit increased susceptibility to oxidation, due to a balanced proportion of *ω*6 and *ω*3 FAs. These findings align with previous studies highlighting the pivotal role of LDL fatty acid composition in modulating oxidative susceptibility, where PUFAs tend to increase oxidative potential, whereas MUFAs are considered protective against oxidation [44,45]. Specifically, *ω*6-FAs, such as AA and LA, are more prone to oxidation, making their balance with *ω*3 FAs critical in modulating oxidative vulnerability [26]. Moreover, it has been suggested that LDL enriched in EPA and DHA exhibit lower oxidative susceptibility [46]. In our study, we observed a higher concentration and relative abundance of AA but also of EPA, leaving a balance between the FAs types. In addition, high levels of oleic acid, which is known for its high resistance to oxidation [26], were detected in participants with HF, potentially contributing to greater protection against LDL oxidation. Consistent with our findings, previous studies have reported similar levels of myristic acid [26] and stearic acid [24,26] between hyperlipidemic and normolipidemic individuals. Given that LDL oxidation is a pivotal trigger for endothelial dysfunction and vascular inflammation, the preserved fatty acid balance and increased antioxidant MUFAs and EPA observed in our FH cohort may contribute to mitigating early endothelial injury. This suggests that dietary modulation of LDL fatty acid composition could support vascular homeostasis and delay the onset of endothelial dysfunction commonly associated with atherogenesis [47].

Thus, although individuals with FH are predisposed to premature atherosclerosis, these data suggest that when LDL fatty acid composition is modulated by a healthy diet, oxidative and pro-inflammatory contributions associated with fatty acids may be attenuated. Evidence supports that Mediterranean diet adherence in FH patients is linked to improved lipid and inflammatory profiles, providing a metabolic-vascular environment less prone to FA-driven oxidative damage [48]. This finding indicates that, while elevated LDL cholesterol remains the primary driver of atherosclerosis in FH, fatty acid-mediated oxidative or inflammatory pathways may be less critical in diet-controlled patients, highlighting the importance of dietary intervention in modulating vascular risk.

When comparing individuals with FH who have or have not experienced a CVE with non-FH individuals, we observed that differences in LDL-FA composition appeared more pronounced in hypercholesterolemic individuals who have had an event. Previous studies have demonstrated that patients with CVD tend to have higher circulating levels of SFAs and trans FAs, both associated with increased risk of atherosclerosis and adverse CVEs. In contrast, levels of *ω*3-PUFAs, particularly EPA and DHA, are often lower in CVD patients, and higher levels are associated with reduced risk of fatal events and improved vascular function [49,50]. Although these findings differ from ours, results from both studies underscore those individuals who have experienced a CVE may exhibit distinct alterations in FA metabolism. These discrepancies may be attributed to population-specific characteristics, as participants in our study were individuals with HF. Finally, it is noteworthy to indicate that our results show no evidence that FA metabolism is further compromised when comparing hypercholesterolemic individuals with and without a history of CVE.

Previous studies have reported that FH affects both the quantity and quality of HDL particles, thereby impairing their role in reverse cholesterol transport [51]. Evidence indicates that HDL quality, rather than HDL quantity, is key to its protective role in CVD [52,53]. However, studies characterizing FA profile of HDL in specific populations, such as individuals with genetic background of FH, remain limited. In our study, we found that, at the class level, individuals with FH were distinguished by a higher proportion of MUFAs, while displaying a similar pattern of PUFAs and SFAs. These small differences can be attributed to the fact that both groups had a similar pattern of FA intake. The comparable SFA balance observed between both groups suggests that the antioxidant capacity of HDLs may not be compromised in FH. This is particularly relevant given that, in the context of FH, SFAs have been reported to increase HDL surface rigidity, thereby potentially impairing their antioxidant function [54]. Conversely, enrichment of HDL with MUFAs, such as oleic acid, makes HDL less susceptible to oxidative modification, independent of antioxidant vitamin content [55].

Additionally, our results suggest that HDL functionality related to FA composition is not compromised in these individuals with HF. These results are supported by the fact that higher levels of more oxidizable FAs, such as AA and LA, have previously been associated with impaired HDL functionality [27,28]. In our study we observed a higher concentration and abundance of AA, alongside with a lower relative abundance of LA in the FH group. Furthermore, the presence of EPA in HDL has been shown to enhance this lipoprotein functionality by influencing their size, lipid composition, and associated enzyme activities, thereby contributing to cardiovascular risk reduction [56] and we detected the EPA in higher concentrations and abundance in the HDLs from FH subjects.

A trend similar to that observed in LDLs was found in the HDL, indicating that subjects with FH and a history of CVE tend to exhibit greater differences in their FA profile compared with healthy individuals. However, the comparison among individuals with FH revealed only limited differences in the FA profile, although those with a history of CVEs exhibited higher levels of DHA and a trend toward increased EPA abundance. This pattern may be partly explained by the greater dietary intake of these FAs reported by this group, suggesting a direct influence of diet on HDL lipid composition.

Finally, the evaluation of differences in FA metabolism revealed that, in LDLs, oleic acid, γ-linolenic acid, α-linolenic acid, AA, and EPA emerged as potential differentiating markers, whereas in HDLs, oleic acid, γ-linolenic acid, AA, and EPA were identified. In both lipoproteins, however, our results indicate that SFAs do not serve as reliable indicators to discriminate metabolic differences between individuals with FH and healthy controls. A previous study demonstrated that hypercholesterolemic patients exhibit significant changes in serum concentrations of palmitoleic acid, oleic acid and DHA. The degree of mono- and polyunsaturation in serum FAs is also altered, with these unsaturated FAs serving as potential biomarkers for hyperlipidemia and reflecting changes in FA metabolism [57]. These results, although obtained in serum, are consistent with our findings showing that unsaturated FAs serve as biomarkers of metabolic differences in FH.

In this context, new findings gave evidence that triglyceride-driven metabolic stress correlates with arterial stiffness and early vascular injury, even in non-diabetic individuals [58]. While our study does not specifically investigate triglyceride-rich lipoproteins or TG–HDL interactions, this underscores the growing recognition of the role of broader lipid-induced metabolic stressors in vascular dysfunction. Our observed maintenance of HDL quality and FA-related functionality in FH supports the notion that dietary and metabolic factors influencing lipid composition might influence early vascular health, beyond traditional cholesterol metrics.

Clinical studies in humans have documented altered serum and tissue FA profiles under statin therapy, including increased concentrations of AA and subtle effects on n-3 long-chain PUFAs [22,59]. However, statin-mediated effects vary by statin type. While mechanistic research shows simvastatin may enhance conversion of LA and EPA into long-chain derivatives [60], most FH participants in our study received atorvastatin, which has demonstrated distinct and less favorable effects on circulating omega-3 fatty acid levels [61]. Therefore, the higher EPA levels in FH participants likely reflect greater dietary intake of omega-3-rich foods, consistent with the above documented adherence to a Mediterranean dietary pattern in this population. This highlights the crucial role of diet in modulating FA profiles in statin-treated populations and suggests factors beyond statin use contribute to the observed EPA enrichment. Recent interventional evidence also demonstrates that LDL-C lowering therapies, such as those interfering with PCSK9 mRNA expression, can improve vascular stiffness [62], suggesting that lipidomic remodeling associated with atherosclerosis may be reversible. This underscores the clinical relevance of detailed FA profiling for assessing metabolic-vascular health and therapy response in FH individuals. Taken together, these findings position FA composition not only as a biomarker of dietary and pharmacologic influence but also as a potential target for monitoring vascular risk modification.

The are several limitations to this study that merit consideration. First, the relatively small sample size (45 participants in total, with only 30 individuals with FH), may limit the generalizability of the findings to the general population with FH. Second, the cross-sectional observational design precludes establishing causal relationships between FH and alterations in the LDL and HDL FA profiles. Third, the assessment of FA intake using a food frequency questionnaire (FFQ) may be subject to recall bias and potential misestimation of dietary intake. Fourth, differences in sex and age distribution across groups may have influenced some clinical and biochemical outcomes, including the higher systolic blood pressure observed particularly in the FH-CVE subgroup, a difference likely reflecting the older age distribution in this group. Finally, although we did not apply formal corrections for multiple comparisons or perform multivariable adjustments due to the exploratory nature and limited sample size of this study, these limitations should be considered when interpreting the data. Nevertheless, these findings emphasize the need for future larger and longitudinal studies with comprehensive statistical modeling to validate these observations.

## 5. Conclusions

In conclusion, our findings demonstrate that FH alters FA metabolism, with the most pronounced changes occurring in LDL particles, whereas HDL is only modestly affected. The greatest metabolic differences compared with non-FH relatives were observed in FH individuals with a history of CVEs; however, within the FH population, a history of CVEs did not appear to significantly influence FA metabolism. Unsaturated FAs emerged as potential markers for differentiating FA metabolic profiles in FH, underscoring their potential utility as indicators of lipid remodeling and metabolic status in this high cardiovascular risk population.

## Figures and Tables

**Table 1:** Clinical characteristics of participants.

	Non-FH (n = 15)		FH		*p* Value			
		Total (n = 30)	Non-CVE (n = 15)	CVE (n = 15)	Non-FH vs. Total	Non-FH vs. Non-CVE	Non-FH vs. CVE	Non-CVE vs. CVE
Female N, (%)	**7 (46.7)**	**8 (24.2)**	**5 (33.3)**	**3 (20.0)**	*0.180*	*0.456*	*0.121*	*0.409*
Age (years)	**44.5** [39.0–58.0]	**59.0** [46.0–64.0]	**55.0** [40.0–64.0]	**61.0** [56.0–64.0]	***0.009***	*0.220*	***0.001***	*0.095*
***Anthropometric variables***								
Weight (kg)	**74.0** [67.0–81.0]	**79.5** [67.0–93.0]	**87.0** [63.0–94.0]	**75.0** [67.0–88.0]	*0.166*	*0.114*	*0.430*	*0.798*
BMI (kg/m^2^)	**26.8** [24.7–27.9]	**28.5** [25.7–31.6]	**30.3** [25.0–31.6]	**28.4** [25.7–31.6]	*0.132*	*0.170*	*0.229*	*0.783*
WC male (cm)	**103.5** [93.5–106.0]	**102.5** [93.0–109.0]	**102.5** [90.0–109.0]	**103.0** [96.0–109.5]	*0.881*	*0.982*	*0.777*	*0.506*
WC female (cm)	**86.0** [83.0–90.0]	**90.0** [81.0–93.5]	**85.0** [77.0–87.0]	**93.0** [93.0–94.0]	*0.632*	*0.614*	*0.083*	*0.214*
***Blood pressure***								
SBP (mmHg)	**121.5** [111.5–126.5]	**137.8** [125.5–155.0]	**136.0** [107.0–153.0]	**142.5** [132.0–155.5]	***0.018***	*0.237*	***0.004***	*0.254*
DBP (mmHg)	**78.0** [71.0–86.0]	**78.5** [69.0–88.5]	**77.0** [73.5–91.0]	**80.0** [67.0–85.5]	*0.798*	*0.467*	*0.783*	*0.340*
***Lipid profile***								
TAG (mg/dL)	**101.0** [77.0–163.0]	**95.0** [72.0–109.0]	**86.0** [72.0–107.0]	**100.0** [69.0–115.0]	*0.387*	*0.371*	*0.560*	*0.628*
TC (mg/dL)	**217.0** [184.0–265.0]	**223.0** [204.0–251.0]	**234.0** [205.0–280.0]	**212.0** [192.0–251.0]	*0.783*	*0.451*	*0.806*	*0.275*
HDLc (mg/dL)	**50.0** [48.0–65.0]	**49.0** [41.0–53.0]	**49.5** [41.0–58.0]	**47.0** [39.0–53.0]	*0.240*	*0.613*	*0.139*	*0.238*
LDLc (mg/dL)	**146.8** [114.6–183.4]	**152.8** [134.6–176.6]	**157.8** [137.4–188.6]	**151.2** [123.2–76.6]	*0.732*	*0.505*	*0.967*	*0.377*
Apo B (mg/dL)	**103.0** [85.0–128.0]	**120.0** [102.0–140.0]	**119.5** [105.0–140.0]	**120.0** [91.0–141.0]	*0.282*	*0.182*	*0.602*	*0.568*
Apo A1 (mg/dL)	**144.0** [127.0–182.0]	**133.0** [121.0–142.0]	**135.0** [123.0–152.0]	**131.9** [119.0–140.4]	*0.066*	*0.205*	*0.063*	*0.419*
Lp(a) (mg/dL)	**19.5** [7.9–45.5]	**26.1** [14.7–64.4]	**26.9** [14.7–57.8]	**24.4** [14.9–69.1]	*0.365*	*0.571*	*0.332*	*0.830*
***Biochemical parameters***								
CRP (mg/L)	**2.1** [1.0–2.6]	**2.0** [0.9–5.1]	**1.4** [0.8–2.9]	**4.1** [1.0–5.3]	*0.878*	*0.431*	*0.309*	*0.230*
Glucose (mg/dL)	**89.0** [79.0–92.0]	**91.0** [84.0–99.0]	**91.0** [84.0–97.0]	**86.0** [83.0–107.0]	*0.394*	*0.187*	*0.846*	*0.658*

Values are shown as median [IQR]. Median values were compared with the Wilcoxon Mann–Whitney test. Proportion of men and women were compared with chi-square (χ^2^) test. *p-values are shown in italic and p-values* < 0.05 were considered significant and highlighted in bold. FH: familial hypercholesterolemia; CVE: cardiovascular event; NS: non-smoker; S: smoker; ES: ex-smoker; BMI: body mass index; WC: waist circumference; SBP: systolic blood pressure; DBP: diastolic blood pressure; TAG: triglycerides; TC: total cholesterol; HDLc: high-density lipoprotein cholesterol; LDLc: low-density lipoprotein cholesterol; Apo: apolipoprotein; Lp(a): lipoprotein (a); CRP: C-reactive protein.

**Table 2:** Dietary intake of fatty acids assessed by FFQ.

	Non-FH (n = 15)	FH			*p* Value			
		Total (n = 30)	Non-CVE (n = 15)	CVE (n = 15)	Non-FH. vs. Total	Non-FH vs. Non-CVE	Non-FH vs. CVE	Non-CVE vs. CVE
***SFAs* (g/day)**	**19.3** [17.2–32.8]	**15.6** [12.4–22.4]	**16.7** [13.4–22.0]	**14.4** [11.6–24.9]	*0.061*	*0.089*	*0.139*	*0.885*
Myristic acid	**1.4** [1.0–2.7]	**1.1** [0.6–1.5]	**1.0** [0.8–1.2]	**1.1** [0.5–2.1]	*0.072*	*0.073*	*0.218*	*0.707*
Palmitic acid	**10.6** [9.6–17.9]	**8.8** [7.3–13.2]	**9.3** [7.8–11.7]	**8.2** [7.0–13.6]	*0.074*	*0.121*	*0.139*	*0.839*
Stearic acid	**3.7** [3.4–7.2]	**2.8** [2.2–4.5]	**3.1** [2.3–4.3]	**2.5** [2.1–4.8]	***0.027***	***0.042***	*0.090*	*0.977*
***MUFAs* (g/day)**	**35.5** [27.1–46.9]	**35.1** [24.8–42.8]	**29.5** [24.9–41.0]	**35.2** [19.4–43.8]	*0.304*	*0.332*	*0.443*	*0.931*
Oleic acid	**1.1** [0.9–1.9]	**1.0** [0.8–1.1]	**0.9** [0.8–1.1]	**1.0** [0.8–1.3]	*0.096*	*0.052*	*0.412*	*0.385*
***PUFAs* (g/day)**	**32.5** [24.6–42.9]	**32.3** [22.7–39.5]	**27.7** [22.9–38.9]	**33.2** [16.5–40.1]	*0.449*	*0.497*	*0.547*	*0.977*
Linoleic acid	**11.3** [9.4–13.9]	**9.5** [7.1–11.9]	**9.1** [7.3–12.1]	**10.0** [6.9–11.6]	*0.069*	*0.109*	*0.139*	*0.707*
AA	**8.5** [6.0–10.9]	**5.9** [4.9–8.7]	**5.5** [4.9–8.7]	**6.1** [4.9–8.7]	*0.061*	*0.109*	*0.112*	*0.839*
Linolenic acid	**0.59** [0.53–0.81]	**0.55** [0.46–0.66]	**0.54** [0.47–0.59]	**0.64** [0.43–0.74]	*0.198*	*0.109*	*0.642*	*0.259*
EPA	**0.10** [0.09–0.19]	**0.09** [0.06–0.11]	**0.07** [0.05–0.11]	**0.09** [0.07–0.14]	*0.051*	***0.020***	*0.366*	*0.132*
DHA	**0.13** [0.12–0.16]	**0.19** [0.12–0.24]	**0.14** [0.11–0.19]	**0.23** [0.14–0.37]	*0.051*	*0.452*	***0.007***	***0.034***
***TRANS FA* (g/day)**	**0.8** [0.5–1.7]	**0.5** [0.2–1.0]	**0.5** [0.2–1.0]	**0.4** [0.1–2.0]	*0.055*	*0.081*	*0.126*	*0.935*
***SFA* (%)**	**10.0** [7.9–13.8]	**8.8** [7.2–10.5]	**8.9** [7.3–10.6]	**8.0** [6.8–10.5]	*0.062*	*0.217*	*0.050*	*0.513*
***MUFA* (%)**	**16.0** [13.9–19.1]	**16.2** [13.2–20.2]	**16.3** [14.6–20.2]	**14.8** [10.1–20.4]	*0.896*	*0.595*	*0.436*	*0.267*
***PUFA* (%)**	**5.0** [4.5–5.4]	**4.7** [3.9–5.7]	**5.0** [4.2–5.9]	**4.0** [3.4–5.7]	*0.411*	*1.000*	*0.149*	*0.174*

Values are presented as median [IQR]. Comparison between individual fatty acids was assessed with the Mann–Whitney test. *p*-values are shown in italic and *p*-values < 0.05 were considered significant and highlighted in bold. FFQ: food frequency questionnaire; AA: arachidonic acid; EPA: eicosapentaenoic acid: DHA: docosahexaenoic acid; SFAs: saturated fatty acids; MUFAs: monounsaturated fatty acids; PUFAs: polyunsaturated fatty acids. FH: familial hypercholesterolemia; CVE: cardiovascular event.

**Table 3:** Comparison of the concentration of individual and class fatty acids in LDL.

µg FA/mg Protein	Non-FH (n = 15)	FH			*p* Value			
		Total (n = 30)	Non-CVE (n = 15)	CVE (n = 15)	Non-FH vs. Total	Non-FH vs. Non-CVE	Non-FH vs. CVE	Non-CVE vs. CVE
***SFAs***	**479.8** [320.1–636.9]	**395.3** [248.8–595.0]	**363.6** [273.1–595.0]	**410.2** [184.3–666.8]	*0.854*	*0.683*	*0.926*	*0.861*
Myristic acid	**53.5** [26.6–155.6]	**35.7** [25.2–124.2]	**37.9** [22.4–124.2]	**32.1** [29.2–127.9]	*0.54*	*0.436*	*0.798*	**0.56**
Palmitic acid	**255.1** [156.3–321.0]	**241.9** [127.4–296.9]	**237.8** [152.0–290.4]	**244.4** [92.2–337.5]	*0.78*	*0.683*	*0.958*	*0.861*
Stearic acid	**139.5** [80.6–215.3]	**112.4** [77.4–191.6]	**103.4** [79.6–187.0]	**119.9** [62.1–242.1]	*0.836*	*0.653*	*0.926*	*0.798*
***MUFAs***	**467.9** [270.5–560.7]	**628.3** [420.0–761.8]	**530.5** [301.3–786.2]	**635.3** [426.3–761.8]	***0.049***	*0.217*	***0.032***	*0.645*
Oleic acid	**467.9** [270.5–560.7]	**628.3** [420.0–761.8]	**530.5** [301.3–786.2]	**635.3** [426.3–761.8]	***0.049***	*0.217*	***0.032***	*0.645*
***PUFAs***	**1808.5** [1420.2–2173.6]	**2265.5** [1765.2–3036.5]	**2269.5** [1610.3–3036.5]	**2261.5** [1765.2–3050.6]	***0.032***	*0.089*	***0.049***	*0.798*
*ω*6								
Linoleic acid	**920.3** [811.4–1087.9]	**961.8** [686.6–1181.5]	**1087.4** [686.6–1338.4]	**850.7** [666.9–1116.6]	*0.572*	*0.285*	*0.926*	*0.506*
γ-linolenic acid	**153.1** [81.3–230.8]	**263.0** [160.7–405.3]	**245.0** [158.1–438.9]	**267.8** [160.7–405.3]	***0.002***	***0.019***	***0.004***	*0.798*
AA	**439.7** [299.6–616.1]	**759.1** [503.2–1001.5]	**705.9** [445.0–1001.1]	**937.3** [503.2–1070.3]	***0.003***	***0.041***	***0.002***	*0.263*
*ω*3								
α-linolenic acid	**2.4** [1.2–3.1]	**2.6** [2.3–3.7]	**2.6** [1.6–4.5]	**2.6** [2.4–3.7]	***0.04***	*0.149*	***0.04***	*0.798*
EPA	**16.7** [10.8–32.9]	**55.3** [27.6–65.3]	**41.8** [19.8–59.2]	**62.1** [48.5–121.4]	***0.008***	*0.081*	***0.005***	*0.066*
DHA	**165.3** [132.1–293.3]	**175.0** [111.8–251.3]	**174.1** [89.0–251.3]	**175.8** [115.7–375.6]	*0.986*	*0.653*	*0.616*	*0.245*
*ω*6/*ω*3	**7.3** [4.3–9.3]	**8.7** [6.9–10.4]	**9.2** [7.5–12.4]	**7.2** [5.4–9.7]	*0.308*	*0.106*	*0.894*	*0.072*

Values are presented as median [IQR]. Comparison between individual fatty acids per sample was assessed with the Mann–Whitney test. *p*-values are shown in italic and *p*-values < 0.05 were considered significant and highlighted in bold *p* < 0.05 was considered significant. LDL: low-density lipoprotein; AA: arachidonic acid; EPA: eicosapentaenoic acid: DHA: docosahexaenoic acid; SFAs: saturated fatty acids; MUFAs: monounsaturated fatty acids; PUFAs: polyunsaturated fatty acids; FH: familial hypercholesterolemia; CVE: cardiovascular event.

**Table 4:** Comparison of the concentration of individual and class fatty acids in HDL.

µg FA/mg Protein	Non-FH (n = 15)	FH			*p* Value			
		Total (n = 30)	Non-CVE (n =15)	CVE (n = 15)	Non-FH vs. Total	Non-FH vs. Non-CVE	Non-FH vs. CVE	Non-CVE vs. CVE
***SFAs***	**293.8** [130.5–370.3]	**309.5** [182.8–443.2]	**287.5** [199.2–477.5]	**316.5** [156.8–398.5]	*0.424*	*0.412*	*0.595*	*0.775*
Myristic acid	**56.8** [15.3–74.7]	**57.7** [15.0–84.0]	**57.9** [23.6–96.3]	**57.5** [12.2–79.6]	*0.592*	*0.461*	*0.870*	*0.653*
Palmitic acid	**93.5** [76.1–130.7]	**109.8** [87.6–144.4]	**111.6** [85.6–165.7]	**108.8** [87.6–144.4]	*0.497*	*0.513*	*0.624*	*0.806*
Stearic acid		**120.0** [61.3–193.4]	**105.7** [79.8–200.5]	**125.1** [57.3–193.4]	*0.397*	*0.412*	*0.539*	*0.870*
***MUFAs***	**127.2** [83.1–152.2]	**170.2** [115.1–194.7]	**163.4** [95.5–205.8]	**171.5** [166.6–189.7]	***0.003***	*0.126*	***0.000***	*0.461*
Oleic acid	**127.2** [83.1–152.2]	**170.2** [115.1–194.7]	**163.4** [95.5–205.8]	**171.5** [166.6–189.7]	***0.003***	*0.126*	***0.000***	*0.461*
***PUFAs***	**630.3** [427.9–859.3]	**771.8** [518.4–1004.2]	**724.7** [466.1–874.3]	**833.9** [641.4–1098.1]	*0.065*	*0.325*	***0.030***	*0.267*
*ω*6								
Linoleic acid	**272.4** [197.2–314.8]	**269.0** [191.6–329.7]	**262.4** [173.4–359.9]	**271.8** [191.6–329.7]	*0.766*	*0.806*	*0.806*	*0.935*
γ-linolenic acid	**25.9** [9.9–45.9]	**47.4** [25.9–81.9]	**42.2** [25.9–90.4]	**48.2** [23.4–81.9]	***0.013***	***0.045***	***0.026***	*0.713*
AA	**163.0** [108.3–259.8]	**264.4** [202.1–384.9]	**248.0** [165.0–282.3]	**340.0** [220.3–411.9]	***0.006***	*0.089*	***0.003***	*0.149*
*ω*3								
α-linolenic acid	**0.4** [0.2–0.5]	**0.5** [0.4–0.7]	**0.4** [0.3–0.6]	**0.5** [0.4–0.8]	*0.069*	*0.233*	*0.056*	*0.250*
EPA	**6.8** [4.1–10.3]	**16.4** [8.4–24.3]	**10.4** [6.3–22.3]	**19.4** [ 11.2–41.6]	***0.012***	*0.217*	***0.002***	*0.061*
DHA	**100.9** [86.4–145.8]	**132.4** [85.3–167.0]	**99.1** [62.4–155.7]	**146.6** [96.6–194.9]	*0.397*	*0.935*	*0.116*	*0.068*
*ω*6/*ω*3	**3.3** [2.7–5.5]	**4.6** [3.4–5.4]	**5.3** [4.7–5.6]	**3.6** [2.9–4.6]	*0.256*	*0.106*	*0.744*	***0.007***

Values are presented as median [IQR]. Comparison between individual fatty acids per sample was assessed with the Mann–Whitney test. *p*-values are shown in italic and *p*-values < 0.05 were considered significant and highlighted in bold. HDL: high-density lipoprotein; AA: arachidonic acid; EPA: eicosapentaenoic acid: DHA: docosahexaenoic acid; SFAs: saturated fatty acids; MUFAs: monounsaturated fatty acids; PUFAs: polyunsaturated fatty acids; FH: familial hypercholesterolemia; CVE: cardiovascular event.

## Data Availability

The information outlined in the manuscript, as well as the code book and analytic code, will be provided upon a reasonable request, subject to scientific approval.

## Supplementary Materials

The following supporting information can be downloaded at: https://www.mdpi.com/article/10.3390/nu18010092/s1, Table S1: Use of lipid-lowering, anti-diabetic, and anti-hypertensive therapies in non-FH and FH subjects; Table S2: Comparison of the relative abundance (%) of the individual fatty acids in LDL; Table S3: Comparison of the relative abundance (%) of the individual fatty acids in HDL.

## Abbreviations

The following abbreviations are used in this manuscript:

AA	Arachidonic acid;
CHD	Coronary heart disease
CVE	Cardiovascular event
DHA	Docosahexaenoic acid
EPA	Eicosapentanoic acid
FH	Familial hypercholesterolemia
LA	Linoleic acid
LFP	Lipoprotein-free plasma
TAG	Triglycerides
TC	Total cholesterol
WC	Waist circumference
