# Supplementary material for: Lipoprotein-Specific Fatty Acid Profiles in Familial Hypercholesterolemia: Associations with Cardiovascular History and Dietary Patterns
Source: Nutrients. 2025 Dec 27;18(1):92. doi: 10.3390/nu18010092 (PMC12787433; doi:10.3390/nu18010092)
Supplement: Supplementary file 1 [file nutrients-18-00092-s001.zip › nutrients-3987450-supplementary.pdf]

A. López-Yerena, R. Arroyo-Olivares[5], V. Santisteban, N. Muñoz-Garcia, R. Estruch, P. Mata, L. Badimon, and T. Padro

Table S1. Use of lipid-lowering, anti-diabetic, and anti-hypertensive therapies in non-FH and FH subjects.

		**FH**		
	**No-FH (n=15)**	**Total (n=30)**	**No-CVE (n=15)**	**CVE (n=15)**
**LLT, n (%)**				
Statins	4 (26.7%)	28 (93.3%)	15 (100%)	13 (86.7%)
Resins	0 (0%)	1 (3.3%)	0 (0%)	1 (6.7%)
Fibrates	2 (13.3%)	0 (0%)	0 (0%)	0 (0%)
Ezetimibe	2 (13.3%)	23 (76.7%)	10 (66.7%)	3 (20%)
LDL apheresis	0 (0%)	2 (6.7%)	0 (0%)	2 (13.3%)
**Anti-diabetic , n (%)**	1 (6,7%)	3 (10.0%)	1 (6.7%)	2 (13.3%)
**Anti-hypertensive , n (%)**	2 (13,3%)	6 (20.0%)	2 (13.3%)	4 (26.7%)

Data are presented as number of subjects and percentage of each group. FH: Familial hypercholesterolemia; LLT: lipid-lowering therapy; FH: familial hypercholesterolemia; CVE: cardiovascular event; LDL: Low-density lipoprotein.

Table S2. Comparison of the relative abundance (%) of the individual fatty acids in LDL.

Fatty acids (%)	No-FH (n=15)	FH			*p values*			
		**Total (n=30)**	**No-CVE (n=15)**	**CVE (n=15)**	**No-FH vs. Total**	**No-FH vs. No-CVE**	**No-FH vs. CVE**	**No-CVE vs. CVE**
SFAs	**19.7** [8.5 - 25.8]	**11.1** [7. 7 - 22.6]	**10.1** [8.8 - 19.9]	**12.0** [6.8 - 22.8]	*0.190*	*0.325*	*0.213*	*0.674*
Myristic acid	**1.9** [1.0 - 5.5]	**1.3** [0.7 - 4.5]	**1.4** [0.5 - 3.8]	**1.1** [0.8 - 4.7]	*0.145*	*0.202*	*0.229*	*0.616*
Palmitic acid	**9.1** [5.4 - 12.9]	**6.5** [4.5 - 11.1]	**6.2** [5.9 - 11.1]	**7.8** [3.5 - 11.6]	*0.216*	*0.367*	*0.229*	*0.674*
Stearic acid	**6.1** [2.3 - 8.8]	**3.2** [2.3 - 6.1]	**3.0** [2.6 - 6.1]	**3.5** [2.3 - 7.0]	*0.244*	*0.325*	*0.319*	*0.991*
MUFAs	**16.1** [12.3 - 20.3]	**16.3** [14.5 - 18.4]	**16.6** [14.5 - 18.4]	**16.0** [14.3 -20.0]	*0.509*	*0.567*	*0.587*	*0.893*
Oleic acid	**16.1** [12.3 - 20.3]	**16.3** [14.5 - 18.4]	**16.6** [14.5 - 18.4]	**16.0** [14.3 - 20.0]	*0.509*	*0.567*	*0.587*	*0.893*
PUFAs	**63.9** [58.4 - 70.2]	**66.6** [62.0 - 74.5]	**66.7** [61.7 - 76.4]	**66.4** [62.0 - 73.3]	*0.120*	*0.174*	*0.198*	*0.645*
ω6								
Linoleic acid	**31.2** [28.2 - 37.8]	**28.6** [24.5 - 33.0]	**32.6** [28.2 - 33.4]	**26.0** [23.9 - 29.0]	***0.032***	*0.683*	***0.001***	***0.005***
γ-linolenic acid	**6.1** [4.4 - 7.0]	**7.5** [6.3 - 10.5]	**7.7** [6.3- 11.2]	**7.4** [6.2 - 9.8]	***0.007***	***0.016***	***0.026***	*0.735*
AA	**16.0** [13.8 - 21.0]	**21.5** [17.0 - 28.9]	**20.3** [15.4 - 25.6]	**26.6** [17.1 - 28.9]	***0.007***	*0.074*	***0.005***	*0.361*
ω3								
α-linolenic acid	**0.08** [0.05-0.10]	**0.08** [0.07 - 0.10]	**0.08** [0.08 -0.11]	**0.08** [0.06 - 0.10]	*0.408*	*0.345*	*0.645*	*0.532*
EPA	**0.8** [0.6 - 1.2]	**1.5** [0.9 - 2.3]	**1.1** [0.8 - 1.5]	**2.3** [0.9 - 2.8]	***0.016***	*0.089*	***0.016***	***0.044***
DHA	**6.3** [4.9 - 10.2]	**5.3** [4.3 - 6.9]	**4.9** [3.8 - 6.4]	**5.7** [4.7 - 7.8]	*0.109*	*0.056*	*0.406*	*0.229*

Values are presented as median [IQR]. Comparison between individual fatty acids per sample was assessed with the Mann Whitney-test. $p<0.05$ was considered significant. LDL: Low-density lipoprotein; AA: Arachidonic acid; EPA: Eicosapentaenoic acid: DHA: Docosahexaenoic acid; SFAs: Saturated fatty acids; MUFAs: Monounsaturated fatty acids; PUFAs: Polyunsaturated fatty acids; FH: familial hypercholesterolemia; CVE: cardiovascular event.

Table S3. Comparison of the relative abundance (%) of the individual fatty acids in HDL.

Fatty acids (%)	No-FH (n=15)	FH			*p value*			
		Total (n=30)	No-CVE (n=15)	CVE (n=15)	No-FH vs. Total	No-FH vs. No-CVE	No-FH vs. CVE	No-CVE vs. CVE
SFAs	**28.0** [19.7 - 40.6]	**23.4** [16.1 -34.0]	**27.1** [17.8 -39.3]	**22.7** [14.1 - 27.9]	*0.277*	*0.775*	*0.116*	*0.250*
Myristic acid	**5.5** [1.9 - 7.6]	**4.6** [1.6 - 6.2]	**4.9** [2.0 - 7.0]	**4.1** [1.4 - 5.8]	*0.384*	*0.903*	*0.174*	*0.305*
Palmitic acid	**10.9** [7.8 - 20.1]	**8.6** [7.3 - 16.7]	**8.9** [7.5 - 19.5]	**8.4** [6.6 - 11.1]	*0.209*	*0.513*	*0.137*	*0.513*
Stearic acid	**12.5** [6.9 - 13.9]	**10.1** [5.7 - 13.9]	**11.6** [6.8 - 15.5]	**9.6** [4.8 - 12.1]	*0.411*	*0.870*	*0.106*	*0.202*
MUFAs	**11.4** [10.5 - 15.9]	**12.3** [10.7 - 14.7]	**12.5** [10.2 - 14.3]	**12.1** [10.7 - 15.2]	*0.528*	*0.713*	*0.486*	*0.713*
Oleic acid	**11.4** [10.5 - 15.9]	**12.3** [10.7 - 14.7]	**12.5** [10.2 - 14.3]	**12.1** [10.7 - 15.2]	*0.528*	*0.713*	*0.486*	*0.713*
PUFAs	**60.0** [50.8 - 64.4]	**62.6** [53.6 - 68.3]	**61.3** [47.5 - 66.2]	**62.6** [59.4 - 69.2]	*0.299*	*0.838*	*0.116*	*0.367*
ω6								
Linoleic acid	**26.7** [20.8 - 32.1]	**20.4** [18.7 - 22.8]	**20.7** [18.7 - 29.2]	**20.3** [18.6 - 22.2]	***0.015***	*0.106*	***0.011***	*0.305*
γ-linolenic acid	**3.2** [2.4 - 3.9]	**4.0** [3.1 - 5.1]	**3.8** [3.1 - 4.7]	**4.3** [3.1 - 5.4]	***0.012***	***0.041***	***0.026***	*0.653*
AA	**16.5** [13.7 - 20.2]	**21.8** [16.9 - 27.2]	**19.1** [15.5 - 26.6]	**22.6** [19.6 - 27.3]	***0.004***	*0.074*	***0.001***	*0.217*
ω3								
α-linolenic acid	**0.04** [0.03 - 0.05]	**0.04** [0.03 - 0.05]	**0.04** [0.03 - 0.05]	**0.04** [0.04 - 0.05]	*0.236*	*0.513*	*0.174*	*0.305*
EPA	**0.7** [0.5 - 1.4]	**1.2** [0.8 - 2.3]	**1.1** [0.6 - 1.5]	**1.9** [1.0 - 2.4]	***0.044***	*0.461*	***0.006***	*0.061*
DHA	**12.1** [8.1 - 14.6]	**10.3** [7.9 - 12.0]	**8.1** [7.3 - 10.5]	**11.1** [10.1 - 13.1]	*0.116*	***0.045***	*0.486*	***0.013***

Values are presented as median [IQR]. Comparison between individual fatty acids per sample was assessed with the Mann Whitney-test. $p<0.05$ was considered significant. HDL: High-density lipoprotein; AA: Arachidonic acid; EPA: Eicosapentaenoic acid: DHA: Docosahexaenoic acid; SFAs: Saturated fatty acids; MUFAs: Monounsaturated fatty acids; PUFAs: Polyunsaturated fatty acids; FH: familial hypercholesterolemia; CVE: cardiovascular event.
